# Three‐dimensional genome structure and function

**DOI:** 10.1002/mco2.326

**Published:** 2023-07-08

**Authors:** Hao Liu, Hsiangyu Tsai, Maoquan Yang, Guozhi Li, Qian Bian, Gang Ding, Dandan Wu, Jiewen Dai

**Affiliations:** ^1^ Department of Oral and Cranio‐Maxillofacial Surgery Shanghai Ninth People's Hospital, Shanghai Jiao Tong University School of Medicine College of Stomatology, Shanghai Jiao Tong University National Center for Stomatology National Clinical Research Center for Oral Diseases Shanghai Key Laboratory of Stomatology Shanghai China; ^2^ School of Stomatology Weifang Medical University Weifang China; ^3^ School of Clinical Medicine Weifang Medical University Weifang China; ^4^ Shanghai Institute of Precision Medicine Shanghai China

**Keywords:** cancer, congenital developmental abnormality, three‐dimensional genome, topologically associating domain

## Abstract

Linear DNA undergoes a series of compression and folding events, forming various three‐dimensional (3D) structural units in mammalian cells, including chromosomal territory, compartment, topologically associating domain, and chromatin loop. These structures play crucial roles in regulating gene expression, cell differentiation, and disease progression. Deciphering the principles underlying 3D genome folding and the molecular mechanisms governing cell fate determination remains a challenge. With advancements in high‐throughput sequencing and imaging techniques, the hierarchical organization and functional roles of higher‐order chromatin structures have been gradually illuminated. This review systematically discussed the structural hierarchy of the 3D genome, the effects and mechanisms of cis‐regulatory elements interaction in the 3D genome for regulating spatiotemporally specific gene expression, the roles and mechanisms of dynamic changes in 3D chromatin conformation during embryonic development, and the pathological mechanisms of diseases such as congenital developmental abnormalities and cancer, which are attributed to alterations in 3D genome organization and aberrations in key structural proteins. Finally, prospects were made for the research about 3D genome structure, function, and genetic intervention, and the roles in disease development, prevention, and treatment, which may offer some clues for precise diagnosis and treatment of related diseases.

## Graphical abstract

Linear DNA undergoes a series of compression and folding events, resulting in various three‐dimensional (3D) structural units in mammalian cells. These structures play crucial roles in regulating gene expression, cell differentiation, organ development, and disease progression. Here, the 3D genome structure and function were systematically reviewed.

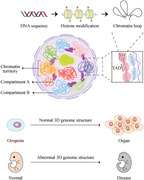

## 1 INTRODUCTION

The three‐dimensional (3D) genome structure plays a crucial role in determining cell fate and maintaining normal cellular functions.^1^ In the late 19th century, the existence of chromatin territory and euchromatin/heterochromatin in the cell nucleus was proposed based on findings from optical microscopes and chromatin dyes,^2^ and Hans Winker introduced the term “genome” in 1920.^3^ With the development of fluorescent in situ hybridization, the individual chromosomal territories could be directly observed.^4^, ^5^ Chromosome conformation capture was first reported in 2002, which employs formaldehyde‐induced chromatin cross‐linking and restriction enzyme digestion to connect spatially proximate DNA fragments.^6^ The interaction frequency between two specific loci in the genome is detected in a “one versus one” format, using polymerase chain reaction or next‐generation sequencing technology.^6^ Circular chromosome conformation capture facilitates the detection of specific DNA fragments' interactions with other genomic regions. Designing primers for the detected fragment achieves a “one versus all” format, allowing for the detection of all genome regions.^7^, ^8^ In 2009, genome‐wide chromosome conformation capture (Hi‐C) was reported and can detect all chromatin interactions throughout the genome, referred to as “all versus all”.^9^ Furthermore, remarkable advancements in optical and electron microscopy technologies have enabled direct observation of chromatin structures. The development of super‐resolution fluorescence imaging techniques, such as stimulated emission depletion and structured illumination microscopy, has been especially notable.^10^ By combining these techniques with specific DNA probes, the chromatin conformation of particular genomic regions within individual cells can be observed, providing new insights for exploring gene expression regulation and other intricate biological processes within the cell nucleus.^11^, ^12^

DNA sequence undergoes a series of complex compressions and folding to ultimately form chromosomes.^13^ A chromosome maintains a specific location within the nucleus, denoted as a chromosome territory (CT).^14^ These chromosomes are organized in a structured manner within the nucleus and can be classified into euchromatin and heterochromatin based on gene transcriptional activity.^15^, ^16^ At the subchromosomal level, A/B compartments, with an average size of 3−5 Mb, correspond to euchromatin and heterochromatin, respectively.^17^, ^18^ Compartments at the megabase level can be further divided into different topologically associating domains (TADs).^19^ TAD represents the fundamental units of 3D genome structure and function.^20^, ^21^, ^22^ High‐frequency interactions between enhancers and promoters within TAD regulate cell‐specific expression of development‐related genes.^22^ The CCCTC‐binding factor (CTCF) at TAD boundaries isolates aberrant regulatory interference, ensuring efficient transcription.^23^ At a smaller scale (<2 Mb), chromatin loops often link enhancers and promoters, playing a crucial role in the regulation of gene transcription.^15^ Structural variations or structural protein abnormalities can lead to disease by disrupting TAD structure and affecting cis‐regulatory element function.^24^, ^25^, ^26^ These may be critical pathological mechanisms for many diseases, such as congenital diseases and cancers.^27^, ^28^

Here, we tried to make a systematic review of the 3D genome structure, function, and relationship with diseases. First, the structural characteristics of mammalian 3D chromatin organization were summarized. Then, the effect and mechanisms of cis‐regulatory elements’ interaction in the3D genome for regulating spatiotemporally specific gene expression, the role and mechanisms of dynamical changes of 3D chromatin conformation during embryonic development were discussed. Subsequently, the pathological mechanisms of some diseases, such as congenital developmental abnormalities and cancer, which attribute to alterations in 3D genome organization and aberrations in key structural proteins, were systematically reviewed. Finally, prospects were made for the research about 3D genome structure, function, and genetic intervention, and the roles in disease development, prevention, and treatment.

## 2 3D GENOME STRUCTURE

**FIGURE 1:**
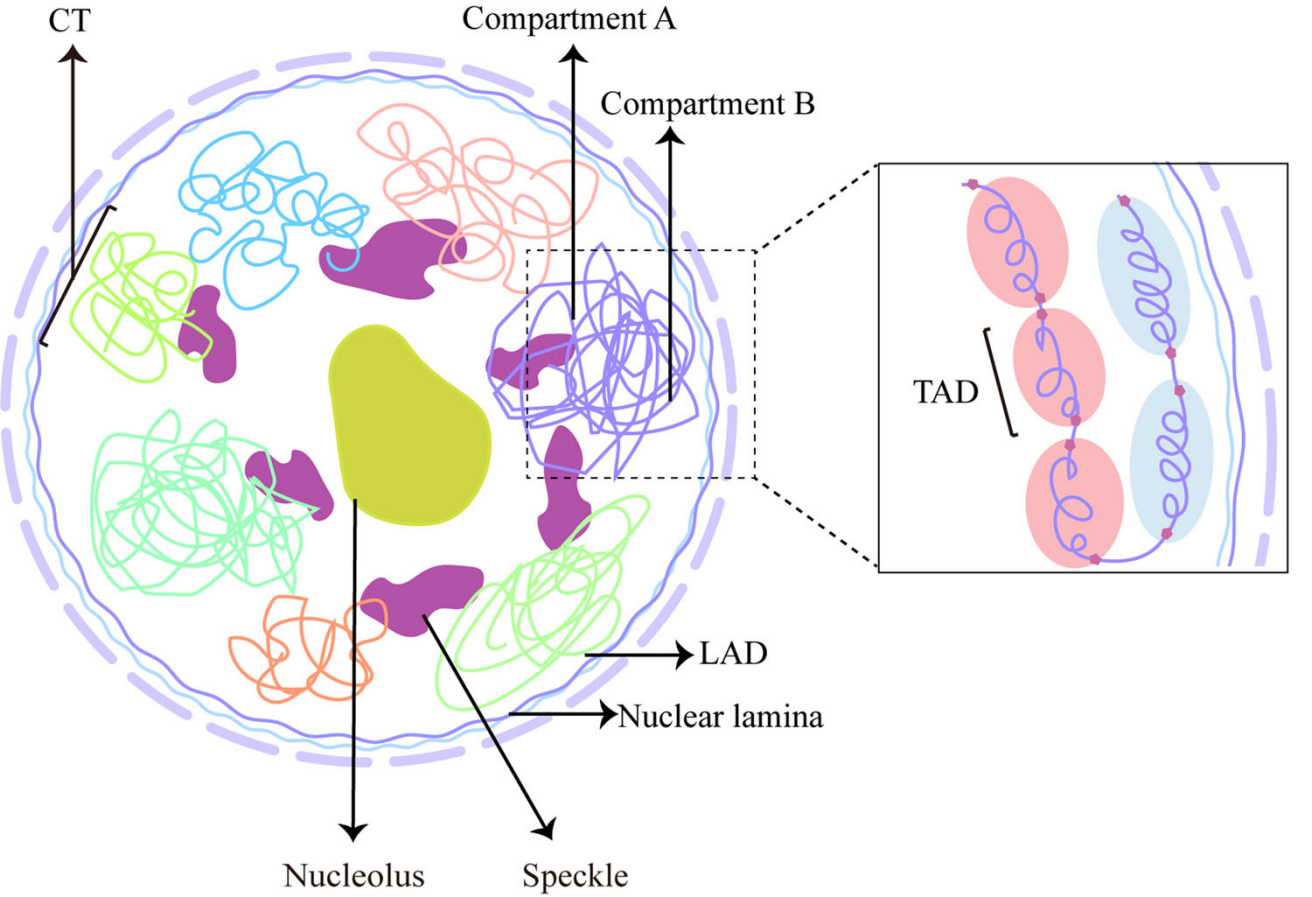
Chromosome 3D structure. In mammalian cells, chromosomes nonrandomly occupy specific regions within the interphase nucleus, known as chromosome territory (CT). Chromosomes can be classified into euchromatin and heterochromatin based on their transcriptional activity and regulatory roles. Euchromatin contains numerous transcriptionally active genes and is loosely arranged, while heterochromatin is tightly organized and primarily comprises transcriptionally silent genes. Heterochromatin at the nuclear periphery interacts with nuclear lamins, giving rise to Lamin‐associated domains (LADs). At the megabase level, the A/B compartment is further subdivided into TAD. Covering approximately 90% of chromatin structure, TAD represents the basic unit of 3D genome function and structure. Within TAD, high‐frequency interactions between enhancers and promoters regulate cell‐specific expression of developmental genes. CTCF, located at TAD boundaries, effectively isolates aberrant regulatory information interference and ensures smooth transcription.

The hierarchical structure of the 3D genome, from high to low levels, includes CT, A/B compartment, topologically associated domain, and chromatin loop (Figure 1).

### 2.1 Chromatin and chromosome territory

In mammalian cells, chromosomes are formed from approximately 2 m of DNA sequences through a series of compressions and folding events.^29^, ^30^, ^31^ Each nucleosome encompasses around 146 base pairs of DNA, giving rise to chromatin fibers that subsequently condense and fold to create chromosomes.^21^, ^32^ These chromosomes nonrandomly occupy specific regions within the interphase nucleus, known as CT.^13^, ^22^ Chromosomes with similar characteristics tend to cluster and are influenced by size, gene density, and transcriptional activity.^33^, ^34^, ^35^ The distribution of chromosome territories can vary among different cell types.^36^, ^37^, ^38^ For instance, differences in nuclear shapes between human fibroblasts and lymphocytes result in distinct radial distributions of chromosome territories.^39^ Moreover, interactions between CT boundaries may be associated with disease‐related chromosomal translocations.^14^

Chromosomes can be classified into euchromatin and heterochromatin based on their transcriptional activity and regulatory roles.^9^ Euchromatin, comprising numerous transcriptionally active genes, exhibits a loosely organized structure, whereas heterochromatin, characterized by transcriptionally silent genes, displays a tightly packed arrangement.^40^ Heterochromatin at the nuclear periphery interacts with nuclear lamins (lamin A/C, B1, and B2), giving rise to Lamin‐associated domains (LADs).^41^, ^42^, ^43^, ^44^ Over 1000 LADs exist in humans and mice, with a median size of approximately 0.5 Mb, comprising 30−40% of the entire genome.^45^, ^46^ LADs are characterized by heterochromatin histone modifications H3K9me2 and H3K9me3 and predominantly exhibit low or silent gene expression.^47^ LADs can be further categorized into constitutive LADs (cLADs) and facultative LADs (fLADs), based on their conservation across cell types.^48^ cLADs retain a relatively stable position in the genome and display high conservation among cell types.^46^ In contrast, fLADs demonstrate variability across cell types, associate with cell type‐specific gene expression regulation and functions, and experience dynamic changes during the cell cycle, cellular differentiation, and circadian rhythms.^49^, ^50^

### 2.2 A/B compartment

At the sub‐chromosomal level, A/B compartments correspond to transcriptionally active and silent chromatin.^51^ These compartmental structures have an average size of 3−5 Mb.^17^ The A compartment is situated near nuclear speckles regions and modified by active histones, while the B compartment locates at the nuclear periphery and is modified by inactive histones.^15^ Sites within the same compartment exhibit more frequent interactions.^15^ Highly spatially plastic, compartments undergo extensive A–B switching during embryonic stem cell differentiation. Genes associated with A–B compartment switching demonstrate increased transcriptional activity throughout development.^16^

### 2.3 Topologically associating domain

At the megabase level, A/B compartments are further divided into different TADs.^52^ In mammalian genomes, TADs tend to be conserved across species and different cell types,^15^, ^53^, ^54^ covering approximately 90% of chromatin structure and serving as the fundamental unit of 3D genome function and structure.^55^ In most cases, TAD formation precedes gene expression.^56^ In the human genome, TADs emerge during the eight‐cell stage when the interactions between regulatory elements within TAD are low and gradually increase throughout embryo development. TADs span approximately 0.1–1 Mb, and up to a maximum of 2 Mb, containing one or more genes.^52^ Smaller sub‐TAD structures reside within TADs and are associated with tissue‐specific gene expression.^57^, ^58^, ^59^ Although earlier studies have suggested that TADs function as a unit at the cell population level, recent studies indicate that TADs are chromatin structures in individual cells, exhibiting globular conformations and sharp structural domain boundaries.^60^, ^61^

TADs boundaries are co‐localized with housekeeping genes, cohesin, and CTCF.^53^, ^54^ CTCF is located at TAD boundaries and effectively compartmentalizes external regulatory information.^53^, ^54^ As a highly conserved zinc finger protein, CTCF plays crucial roles in transcriptional activation/repression, insulation, and the formation of higher‐order chromatin structures.^62^, ^63^ Due to the DNA sequences that bind CTCF being asymmetric, CTCF located at TADs boundaries has directionality.^64^, ^65^ In over 90% of TADs, CTCF sites are situated at boundaries in a convergent orientation.^66^ CRISPR‐edited inversions of CTCF binding sequences disrupt TAD structure and cause abnormal gene expression, suggesting that CTCF orientation is important for TAD.^67^ Multiple CTCF binding sites at TAD boundaries ensure precise gene expression through synergistic and redundant effects.^68^, ^69^, ^70^ Recent studies showed that six CTCF binding sites exist between Epha4 and Pax3 TADs, and are located adjacent to each other. Deletion of a single CTCF binding site can alter Pax3 expression level without causing observable phenotypes.However, deletion of multiple CTCF binding sites leads to TADs fusion and abnormal finger development in mice.^70^ TAD boundaries are highly conserved and remain relatively stable during organ development and tissue differentiation.^16^, ^23^, ^71^ However, human genome analysis suggests that although the vast majority of CTCF loci are conserved, thousands of CTCF loci may be associated with tissue‐specific gene expression.^72^ For instance, CTCF function and insulating activity are positively correlated with the level of enhancer–promoter interactions within TAD.^73^ CTCF at TAD boundaries is more conserved than CTCF located within TAD, with stronger binding, more open chromatin, and lower DNA methylation levels.^74^ Furthermore, TAD boundaries containing a single gene are more conserved than TAD boundaries that contain multiple genes.^75^

### 2.4 Chromatin loop

Chromatin loop is relatively stable, ring‐shaped structure formed by chromatin within 3D space, often containing regulatory elements such as enhancers and promoters.^76^ In 2014, Rao and colleagues discovered approximately 10,000 chromatin loops through the analysis of in situ Hi‐C conducted on human lymphoblastoid cells.^15^ Most of these loops are conservation across various species and cell types.^15^ Genes that form chromatin loops exhibit higher expression levels compared with those did not form chromatin loops, indicating the involvement of chromatin loops in regulating gene expression.^15^ Furthermore, an analysis of 24 cell lines derived from three human germ layers revealed that approximately 28% of chromatin loops varied among different cells.^77^ These tissue‐specific chromatin loops are strongly associated with the tissue‐specific gene expression.^77^

**FIGURE 2:**
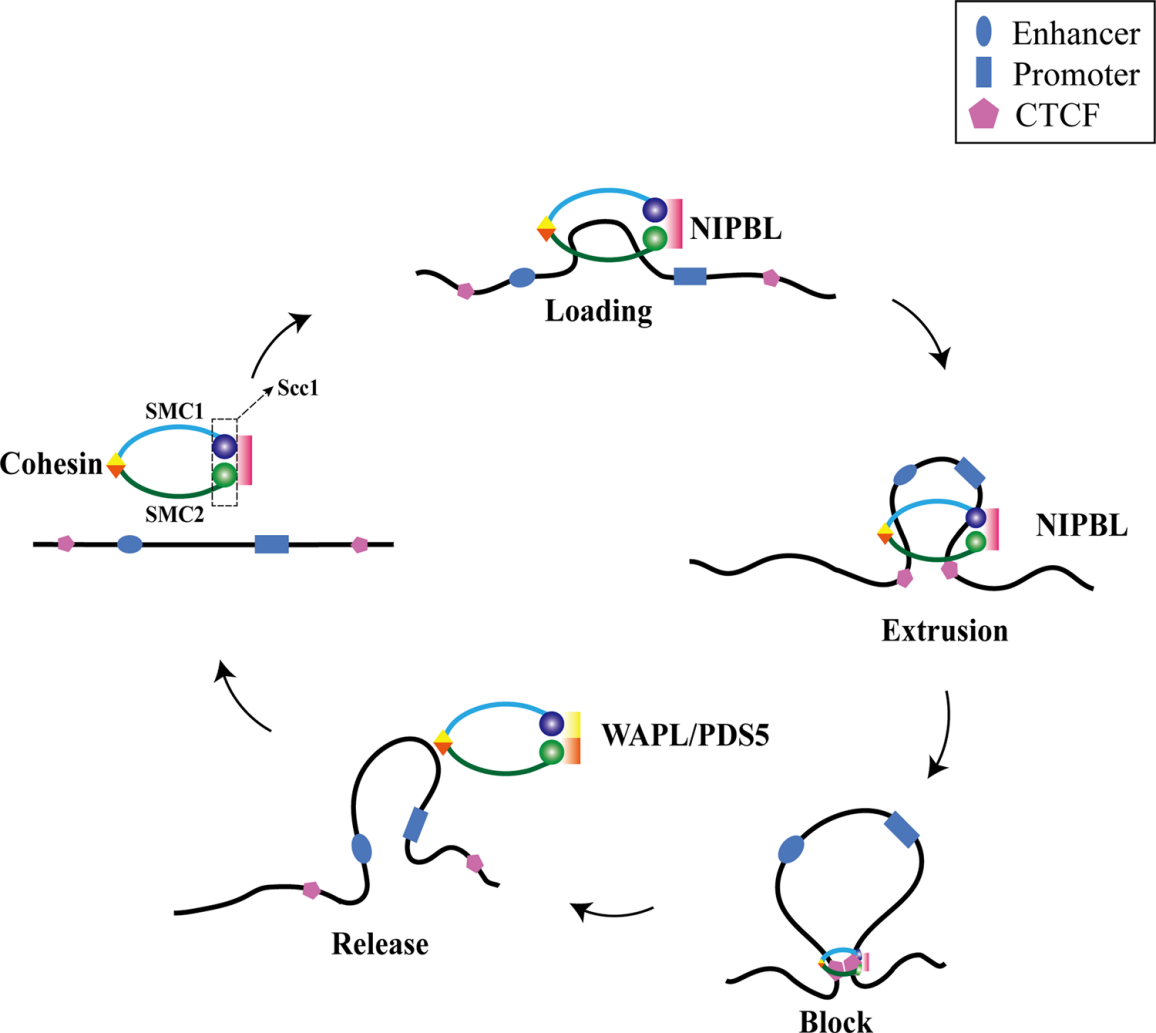
Mechanism of loop extrusion. Cohesin acts as a molecular motor in the “loop extrusion” model: After binding to chromosomes, cohesin moves in two opposite directions along chromatin fibers and extrudes DNA loops until it contacts the target CTCF site in the converging direction. During TAD formation, cohesin undergoes functional changes by interacting with various regulatory factors. The cohesin loading factor, NIPBL, loads cohesin at specific DNA sites and facilitates cohesin's translocation on chromosomal fibers. Cohesin's release from chromosomes requires the PDS5 subunit to recruit the cohesin‐releasing factor, WAPL. The unloading efficiency of WAPL is stronger than that of PDS5, and both participate in the cohesin release process. Finally, cohesin ensures its smooth arrival at the target CTCF site through direct interaction with CTCF.

The “loop extrusion” model, mediated by cohesin‐CTCF cooperation, aptly describes the formation of chromatin loops and TADs^78^ (Figure 2). In this process, CTCF and cohesin exhibit distinct roles.^78^ CTCF acts as a barrier to prevent overextension of cohesin.^79^ Cohesin is a ring‐shaped DNA‐entrapping adenosine triphosphatase (ATPase) complex that works as a molecular motor.^80^ Human cohesin comprises three subunits: SMC1, SMC2, and Scc.^81^ Cohesin moves along the chromatin fiber with a speed of 1.5 kb/s until it interacts with convergently oriented CTCF sites.^82^ This process lasts 10−30 min.^83^ Scc1 plays an essential role in loop extrusion by binding to several regulators, such as NIPBL and PDS5.^84^ The cohesin loading factor NIPBL loads cohesin at specific DNA sites, promoting cohesin translocation across chromosome fibers.^85^, ^86^, ^87^ In addition, NIPBL can activate ATPase to supply energy for the high‐speed movement of cohesin on chromatin fibers.^88^ Cohesin release from chromosomes requires PDS5 to recruit WAPL, a cohesin‐releasing factor.^89^ The unloading efficiency of WAPL is stronger than that of PDS5, and both participate in the process of cohesin release.^89^, ^90^, ^91^ Cohesin ensures its smooth arrival at the CTCF site by directly interacting with CTCF.^92^ The N‐terminal 77‐aa region of CTCF can directly interact with the cohesin Scc1‐SA2, providing a structural and functional basis for the precisely anchoring of cohesin to CTCF binding sites.^93^

The functional changes of the aforementioned cohesin subunits or complexes affect the formation of TAD. For example, deletion of the Scc1 subunit leads to the disruption of TAD,^75^, ^94^ while depletion of WAPL leads to a more than 20‐fold prolongation of the duration of cohesin action, resulting in the formation of a larger chromatin loop, which increases the amount of TAD and/or sub‐TAD.^90^, ^95^, ^96^, ^97^

## 3 MECHANISMS OF 3D GENOME REGULATING GENE EXPRESSION: CIS‐REGULATORY ELEMENTS INTERACTION IN 3D GENOME

**FIGURE 3:**
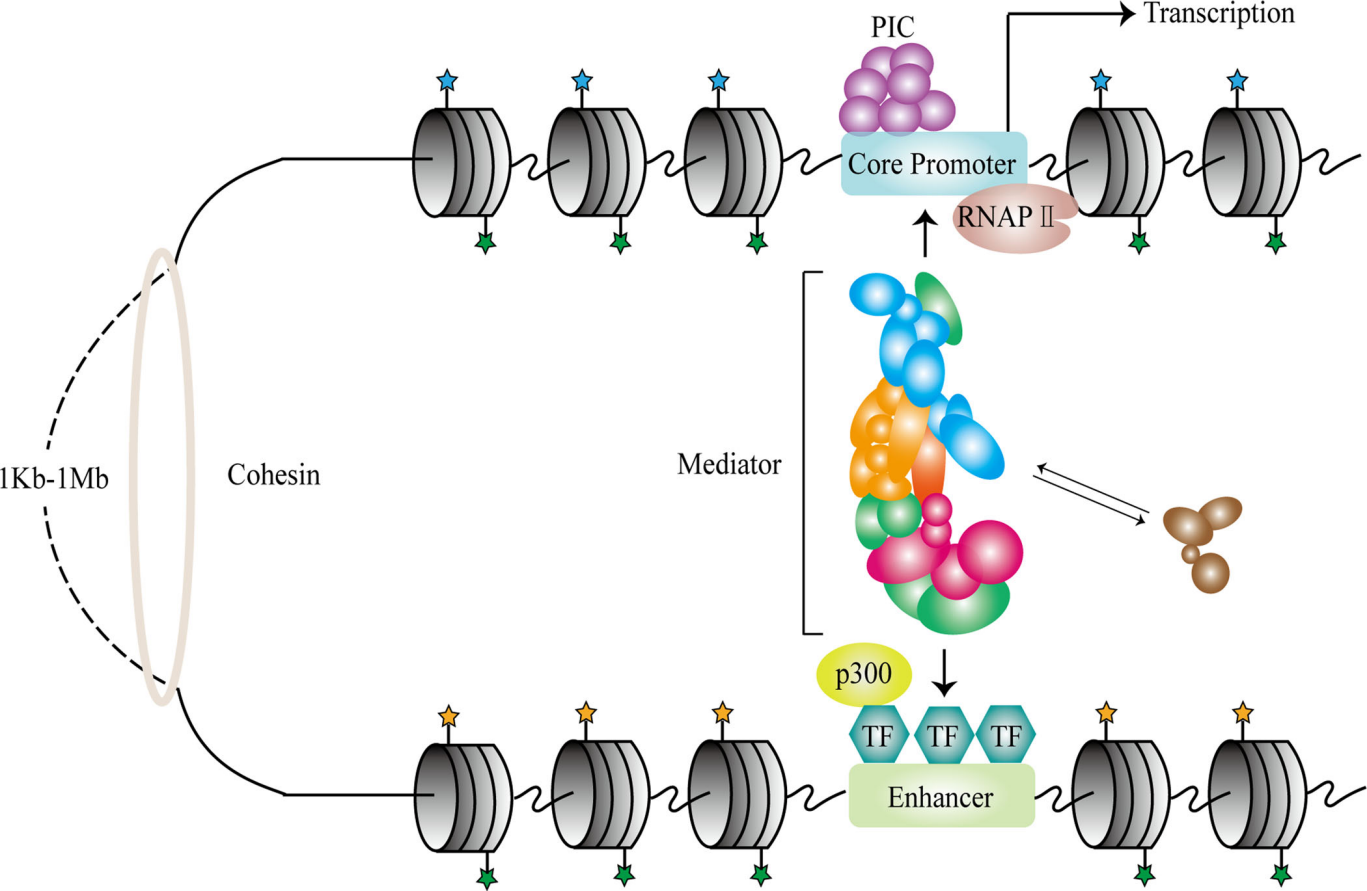
Mechanisms of enhancer–promoter interactions. Within TADs, cis‐regulatory elements play crucial roles in gene expression. Cohesin compresses DNA to form chromatin loops, thereby shortening the distance between enhancers and promoters. The promoter is the transcription start site for mammalian gene expression, determining the location and direction of transcription. The core promoter is a DNA sequence situated approximately 50 bp upstream and downstream of the transcription start site, containing multiple general transcription factor binding sites, and represents the minimal sequence required for initiating gene expression. Enhancers are significant non‐coding elements, typically located in nucleosome‐depleted regions, and contain multiple transcription factor binding sites.

In the human genome, protein‐coding sequences constitute merely 2% of the DNA sequence,^98^ and gene expression is subject to regulation by various noncoding structures, such as promoters and enhancers within the 3D genome, and does not operate in isolation (Figure 3).^99^, ^100^

### 3.1 Promoter

The promoter serves as the starting point for mammalian gene expression and determines the location and direction of transcription.^101^ The core promoter is a DNA sequence located approximately 50 base pairs upstream and downstream of the transcription start site, containing multiple universal transcription factor start sites, and is the minimal sequence that initiates gene expression.^102^, ^103^ The promoter has some transcriptional activity, but it is relatively weak.^104^, ^105^ In specific cases, promoters can act as enhancers to regulate gene expression by interacting with other promoters.^106^, ^107^ The transcription preinitiation complex(PIC) assembles at the promoter and binds to the promoter sequence, a prerequisite for gene transcription.^108^, ^109^ The structure and function of the PIC vary between stages, and the PIC consists of universal transcription factors (TFIIA, TFIIB, TFIID, TFIIE, TFIIF, TFIIH), RNA polymerase II (RNAP II), and other factors in most cases.^110^ These general transcription factors play distinct roles in regulating gene expression, with TFIID recognizing and binding to promoter DNA sequences and TFIIB directing other transcription factors and RNAPII to bind to promoter sequences.^111^ RNAPII undergoes three stages of transcription initiation, pause, and pause release at the promoter,^112^ with RNAPII pausing after about 60 bases of transcription and restarting transcription.^113^ The exact mechanism of this process is unclear, and current studies suggest that RNAPII transcription is influenced by various factors. For example, p300/CBP promotes RNAPII transcription initiation and pause release, whereas TFIID is associated with RNAPII pause.^114^, ^115^

### 3.2 Enhancer

Enhancers are important noncoding elements that regulate the tissue‐specific expression of genes.^116^ Active enhancers typically reside in nucleosome‐deficient regions and contain multiple transcription factor binding sites.^117^, ^118^ The regulatory function of enhancers is generally direction‐independent, with enhancers separated from their cognate promoters by approximately 10^4^−10^6^ base pairs.^119^ Cohesin facilitates enhancer–promoter interactions by shortening the distance between enhancers and promoters through chromatin loop formation.^120^ A single enhancer can act directly with its cognate promoter to regulate target gene expression,^121^ while multiple enhancers can act on the same target gene and regulate its transcription through synergistic or cumulative effects.^122^, ^123^, ^124^ In addition, superenhancers span longer DNA sequences, often containing multiple common enhancers within them, and can drive higher expression levels of target genes with increased activity.^125^ Shadow enhancers are a class of enhancers that partially or completely overlap in expression patterns.^126^ During mammalian development, especially under conditions of physiological or genetic stress, these enhancers with similar expression patterns are able to resist genetic variation and keep relatively stable gene expression levels.^127^ These seemingly “redundant” enhancers provide a guarantee of accurate gene expression.^128^

### 3.3 Mechanisms of enhancer–promoter interactions

Chromatin loops can shorten the distance between enhancers and promoters, but the interaction between them also requires the involvement of transcription factors and multiple cofactors.^129^ Enhancer sequences contain multiple transcription factor binding sites and are able to specifically bind to a variety of transcription factors. Pioneer transcription factors are able to efficiently degrade nucleosomes by binding to DNA sequences through an alpha helix structure,^130^ resulting in a chromatin‐open state.^131^, ^132^

Transcription factors recruit p300/CBP and mediator complexes.^133^ P300/CBP, a transcriptional activator with acetyltransferase activity, directly activates enhancers through histone acetylation.^134^ The mediator complex serves as a functional hub between the enhancer and the promoter and consists of four parts: head, middle, tail, and kinase module (CDK8 module).^135^, ^136^ This complex is capable of transmitting genetic information from the enhancer to the promoter.^137^ First, the tail of the mediator interacts with transcription factors at the enhancer, whereas its head and middle of the mediator are involved in RNAP II recruitment and assembly of the transcriptional preinitiation complex.^138^ The kinase module (CDK8) phosphorylates the carboxy terminus of RNAP II to facilitate transcription initiation.^139^ In conjunction with transcription factors and cofactors, enhancers deliver genetic information to the promoter for precise expression of target genes.

## 4 MECHANISMS OF DYNAMICAL CHANGE OF 3D GENOME AMONG DIFFERENT FUNCTIONAL STATES

The 3D genome undergoes dynamic changes during cell differentiation and organ development to regulate interactions between cis‐regulatory elements, exhibiting diverse functional states. In this section, the mechanisms of these dynamic changes in the 3D genome are summarized.

### 4.1 Chromatin compartment switches

During mammalian cell differentiation, chromatin compartments exhibit dynamic changes at various stages and significantly influence the spatiotemporal‐specific expression of development‐related genes.^140^ Throughout the differentiation of human embryonic stem cells (hESCs), substantial transitions transpire between compartments A and B.^141^, ^142^, ^143^ The number of genomes participating in A–B compartment switches varies among cell types, and the number of TADs involved declines as differentiation progresses.^144^, ^145^ For instance, when ESCs differentiate into skeletal muscle progenitors, approximately 20% of the genome participates in the A–B compartment switches. In contrast, only 6.5% of genomes are involved in compartmentalization when skeletal muscle progenitor cells further differentiate.^144^, ^146^ Among the 10 marker genes associated with preadipocyte differentiation, nine are observed to convert from compartment B to A during differentiation.^147^ Similarly, Prdn1 and Atf4, related to plasma cell fate determination, are transferred to euchromatin, whereas Ebf1, an inhibitor of their differentiation, transitions from euchromatin to heterochromatin.^148^

### 4.2 Dynamical change of TAD

During mammalian development, TAD facilitates precise gene expression by providing a suitable environment while minimizing interference from external regulatory information.^149^ TAD‐D (∼250 kb) and TAD‐E (∼500 kb) are adjacent TADs within the mouse X chromosome inactivation center, containing mutually repressed genes Tsix and Xist, respectively.^54^ Xist is associated with X chromosome inactivation, while Tsix represses the expression of Xist.^150^ In male mice, embryonic stem cell differentiation (mESCs), Tsix is transcriptionally active, but Xist is barely expressing.^151^ In female mESCs, although Tsix is also transcriptionally active, Xist transcript levels gradually increase as mESCs further differentiate, eventually leading to X chromosome inactivation.^152^ In male mESCs, a 40 kb inversion across the TAD‐D and TAD‐E boundaries places the promoters of Tsix and Xist inside each other's TAD, respectively. This alteration in the regulatory environment enables enhancers to interact with nontarget promoters, causing promoters that should regulate Tsix gene expression to be ectopically activated within TAD‐E, ultimately leading to aberrant Xist expression in male mESCs.^56^

The HoxD gene clusters locate between two adjacent TADs: the telomeric side (T‐DOM) and the centromeric side (C‐DOM). During mammalian limb development, HoxD genes are sequentially regulated by spatiotemporal‐specific enhancers within these two TADs.^153^, ^154^, ^155^ The T‐DOM TAD contains multiple enhancers that regulate proximal limb development by controlling the expression of Hoxd9, Hoxd10, and Hoxd11 genes.^156^ In contrast, during subsequent distal limb development, enhancers within T‐DOM are silenced, whereas enhancers within C‐DOM are activated.^157^ Enhancer II1, one of the enhancers in C‐DOM responsible for distal limb development, plays a crucial role in tetrapod distal limb development by binding to the transcription factor Hox13. When the enhancer II1 sequence is inserted into T‐DOM, its activity is almost entirely suppressed, even though the transcription factor Hox13 remains explicitly bound to the sequence within enhancer II1.^158^, ^159^ The regulatory activity of enhancer II1 in the distal limb is restored only after most of the sequence at the mitotic end of T‐DOM (Mtx2‐II1‐T‐DOM) is deleted.^160^ Thus, promoter and enhancer activities relate to the chromatin environment during gene expression.^157^ TADs, as higher‐order chromatin structures, provide an appropriate regulatory environment for enhancer–promoter interactions, ensuring that development‐related genes are expressed in a cell‐ or tissue‐specific manner.

During cell differentiation, sub‐TADs gradually form within TADs, promoting cis‐regulatory elements to precisely regulate target gene expression.^161^, ^162^ Runx1, a transcription factor, is associated with hematopoietic development and is situated within a 1.1 Mb TAD, which forms prior to Runx1 gene expression.^163^, ^164^ Analysis of mESCs reveals the emergence of two sub‐TADs within the Runx1 TAD, promoting tissue‐specific enhancer–promoter interactions.^165^ Similarly, observation of α‐globin protein motifs within erythrocytes at different stages of differentiation in mice shows the appearance of sub‐TADs during cell differentiation.^166^, ^167^

Furthermore, a progressive increase in chromatin accessibility, accompanied by a corresponding rise in the frequency of enhancer–promoter interactions within TADs, is observed during the differentiation of various cell types.^166^ For instance, due to changes in enhancer activity, frequent chromatin loop reconnections are observed during the differentiation from preadipocytes to adipocytes.^168^

## 5 ROLE OF DYNAMICAL CHANGE OF 3D GENOME IN GAMETOGENESIS AND EARLY EMBRYOGENESIS

Meiosis plays a crucial role in germ cell development, facilitating the maturation of sperm and oocytes.^169^, ^170^ Throughout this process, germ cells undergo morphological transformations, and their chromosomes experience intricate 3D structural alterations, ultimately leading to highly differentiated germ cells.^171^ In this section, the dynamic changes of the 3D genome in gametogenesis and early embryogenesis are systematically reviewed.

### 5.1 Role of dynamical change of 3D genome in spermatogenesis

Meiosis I and II enable the development of spermatogonia into spermatozoa,^172^ involving homologous chromosome separation and sister chromatid separation to form round spermatids.^169^, ^173^ Subsequently, these round spermatids undergo a series of complex, dynamic changes to generate mature spermatozoa.^174^ During meiotic prophase, although A/B compartments are still present, TAD disappears due to alterations in cohesin activity in mouse spermatocytes.^175^, ^176^, ^177^ In postmeiotic stages, the level of genomic compartmentalization in round spermatocytes gradually increases. Mature spermatocytes exhibit chromatin higher‐order structures such as compartments and TADs, demonstrating frequent remote interactions between TADs.^178^, ^179^ Comparing Hi‐C data from mature sperm cells, fibroblasts, and ESCs reveals considerable similarity in their 3D genomes.^180^ As protamine replaces over 85% of histone loci in mature spermatozoa, chromatin becomes highly compressed, leading to a more than 10‐fold reduction in sperm cell nuclear volume compared with fibroblasts.^180^ In contrast, retained histones are highly enriched at the promoters of crucial developmental genes. For instance, nucleosomes modified by active histones were observed at the transcription start sites of genes such as EVX1/2 and ID1.^174^, ^181^ Moreover, studies on mouse sperm reveal that mature sperm contain over 10,000 enhancers and over 500 superenhancers.^180^, ^182^ The vast majority of these are identical to those in mESCs, suggesting that regulatory elements are ready for tissue differentiation and cell fate decisions as early as in the sperm.^177^

### 5.2 Role of dynamical change of 3D genome in oogenesis

In contrast to sperm development, oocyte formation begins during the embryonic stage.^183^, ^184^ Primordial germ cells, originating from the proximal epiblast, differentiate into oogenic cells within the embryonic gonads. These oogenic cells undergo a series of differentiations, eventually forming primary oocytes that enter meiosis I prophase. Upon reaching the diplotene stage of meiosis I, the primary oocytes constitute primordial follicles, which are subsequently stored in the ovary. During puberty, the primordial follicles gradually transition hormonally into germinal vesicle oocytes.^185^ These germinal vesicle oocytes continue to undergo hormone‐stimulated meiosis and remain suspended in the metaphase of meiosis II until fertilization.^185^

During oogenesis, the 3D genome structure undergoes a series of dynamic changes, characterized by the gradual disappearance of compartment, TAD, and loop.^186^, ^187^ Meiosis I oocytes and germinal vesicle oocytes exhibit similar numbers of TADs, whereas metaphase II (MII) oocytes display an almost complete absence of TADs and A/B compartments, resulting in a uniform chromatin folding pattern.^188^

### 5.3 Role of dynamical change of 3D genome in zygote

After fertilization, two transcriptionally quiescent gametes fuse to form a zygote, characterized by a more relaxed chromatin structure.^189^, ^190^, ^191^ Transcription remains inactive in zygotes and early embryos.^189^, ^192^ Concurrent with zygotic genome activation (ZGA), gene expression is activated, and an extensive reorganization of the 3D genome structure occurs.^193^, ^194^, ^195^ Mouse embryo studies reveal a progressive increase in TAD intensity and the extent of chromatin remote interactions at the two‐cell stage.^196^, ^197^ In contrast, human embryos exhibit compartments and TADs as early as the eight‐cell stage, with continuous enhancement throughout development.^181^, ^198^ Although the onset of ZGA often coincides with TAD formation in many species, recent research indicates that TAD formation associates with DNA replication and that inhibiting ZGA does not impede TAD formation.^195^

## 6 ROLE OF DYNAMICAL CHANGE OF 3D GENOME IN ORGAN DEVELOPMENT

In addition to playing a crucial role in gametogenesis, dynamic changes in the 3D genome are also important for regulating organ development.

### 6.1 Role of dynamical change of 3D genome in brain development

The mammalian brain, a highly complex organ, is primarily composed of neurons and neuroglial cells.^199^ Glial cells include astrocytes, oligodendrocytes, and microglia.^200^ Neurons can be classified as either excitatory or inhibitory.^201^, ^202^ Apart from microglia, all other cells are derived from neural progenitor cells (NPCs), which strictly regulate the spatiotemporal distribution of gene expression to modulate physiological functions such as memory, cognition, and emotion.^203^, ^204^

During brain development, ESCs first differentiate into NPCs, which subsequently differentiate into neurons and further into various cell subtypes.^199^ Throughout this process, chromatin experiences extensive reorganization and compaction.^205^ Initially, the chromatin of ESCs is globally accessible, but as differentiation progresses, it becomes increasingly condensed, reducing accessibility.^206^ Concurrently, at the compartment level, the A compartment size decreases by approximately 5% compared with ESCs as neural cell differentiation continues.^16^, ^71^ Despite this change, TAD boundaries do not exhibit significant alterations; the TAD landscape remains stable in NPCs, neurons, and glial cells.^207^ As differentiation ensues, TAD sizes undergo a slight increase, interaction levels between TADs in the A compartment decrease, and those in the B compartment increase.^71^

Additionally, human‐specific brain structures, such as the formation of the subplate layer, are associated with the development of human‐specific TADs and chromatin loops.^208^, ^209^ These human‐specific TADs are generally smaller than conserved TADs between species due to chromatin structure alterations, and their boundary CTCF binding strengths are marginally weaker.^208^ Human‐specific enhancers play a crucial role in brain development by effectively regulating target gene tissue‐specific expression.^210^, ^211^, ^212^ For instance, the human‐specific enhancer EOMES controls FGFR2 expression, which is associated with NPCs proliferation.^206^ EPHA7 is regulated by an upstream human‐specific enhancer, and its inactivation impacts the development of human cortical pyramidal cells,^208^ ARHGAP11B collaborates with a human‐specific enhancer located 500 kb away, contributing to the expansion of the human neocortex.^213^

### 6.2 Role of dynamical change of 3D genome in cardiac development

The heart, originating from the mesoderm, has chromatin regulatory mechanisms in its development process that have long captivated researchers. Primarily composed of cardiomyocytes, the heart derives these cells from cardiac progenitor cells, which in turn arise from distinct subgroups of cardiac mesodermal cells.^214^

Cardiomyocyte‐specific compartments form early during heart development and differentiation.^215^ Studies reveal that in adult mouse cardiomyocytes, approximately 66.7% of cardiac‐specific genes reside in compartment A.^216^ During cardiomyocyte differentiation, around 20% of the genome undergoes compartment conversion, with genes transitioning from compartment B to A demonstrating greater cardiac specificity.^217^, ^218^, ^219^ Moreover, as hESCs or human induced pluripotent stem cells (hiPSCs) differentiate into cardiomyocytes, a decrease in the number of TADs, loss of boundaries, and reduction in intensity are observed.^218^ Roughly 70% of TADs remain stable throughout development, suggesting their importance in heart development through the promotion of enhancer–promoter interactions and regulation of developmental gene expression. For instance, Handsdown (Hdn) and Hand2, which are located within the same TAD and closely related to heart development, play a significant role in cardiomyocyte differentiation and heart development.^220^, ^221^ Hdn encodes a lncRNA and modulates Hand2 transcription by inhibiting its upstream enhancer activity. Hdn deficiency results in an abnormal increase in Hand2 expression, leading to the thickening of the right ventricular wall.^222^ Furthermore, structural protein abnormalities associated with TAD formation may cause heart development abnormalities of varying degrees.^214^, ^223^, ^224^ For example, STAG2, one of the cohesin subunits, has its deficiency affecting the proliferation and migration of secondary heart field progenitor cells, leading to delayed heart development and morphological defects.^225^ A heterozygous deletion of the adhesion loading factor NIPBL may induce atrial septal defects.^226^, ^227^

### 6.3 Role of dynamical change of 3D genome in blood system development

The development and differentiation of hematopoietic stem cells (HSCs) provide a valuable model for 3D genomics research. During embryonic development, HSCs initially appear within the principal arteries and subsequently migrate to the liver.^228^ Before birth, HSCs relocate to the bone marrow, where they remain for an extended period. In adult mouse bone marrow, more than 70% of HSCs exist in a quiescent differentiation state.^229^

Studies have demonstrated that as ESCs differentiate into HSCs, the spatial arrangement of TADs experiences dynamic shifts coinciding with the transition of A–B compartments.^230^, ^231^ Within TADs, chromatin accessibility progressively increases, and sub‐TADs form gradually.^165^, ^166^ Furthermore, research on mouse embryos and adult mouse HSCs has revealed that compartments and TADs are predominantly conserved, with a mere 5% of compartments and 12% of TADs undergoing alterations.^232^ In adult mouse HSCs, both compartmentalization and TAD boundary strength augment, whereas chromatin accessibility declines. Approximately 52% of enhancer–promoter interaction levels vary, correlating with differential gene expression.^232^

Erythropoiesis refers to the biological process wherein hematopoietic stem cells and progenitor cells differentiate into mature red blood cells.^233^ Erythroid progenitor cells proliferate and undergo a sequence of morphological transformations, ultimately yielding enucleated reticulocytes.^234^ Throughout erythroid cell differentiation, widespread chromatin condensation occurs, and both chromatin accessibility and transcriptional activity diminish.^235^, ^236^ Heterochromatin significantly compacts, and H3K9me3 is repositioned, leading to numerous long‐range interactions.^237^ Approximately 58% of TADs are disrupted, and TAD boundaries weaken considerably during the final stages of erythroid development. TADs with active chromatin modifications are partially conserved, and GATA1 is thought to participate in the preservation of TADs.^237^

### 6.4 Role of dynamical change of 3D genome in immune system development

B cells and T cells both derive from hematopoietic stem cells in the bone marrow.^238^ Within this environment, common lymphoid cells either differentiate into B cells and innate lymphoid‐like cells or migrate to the thymus, where T cell differentiation is initiated. The development of B and T cells is intimately associated with the recombination of antigen gene loci.

Throughout B cell development, the dimensions, quantity, and positions of the majority of TADs remain relatively constant.^239^ However, significant gene loci associated with B cell fate determination exhibit dynamic changes. The Ebf1 gene locus encodes an essential B cell transport protein and serves a crucial regulatory function in B cell differentiation.^240^ At the pluripotent stage, the Ebf1 locus resides in the transcriptionally repressed B compartment.^241^, ^242^ As cells transition from pre‐pro‐B cells to pro‐B cells, Ebf1 relocates to the transcriptionally active A compartment, playing a role in B cell fate determination.^239^ During the differentiation of B cells into plasma cells, Ebf1 is repositioned to the heterochromatin region.^148^

T cell development relies on Bcl11b expression. In the pluripotent stage, Bcl11b is situated within the B compartment, with its activation closely tied to the non‐coding RNA ThymoD.^243^, ^244^ ThymoD facilitates local DNA demethylation through transcription, promoting CTCF‐DNA binding and cohesin protein recruitment. This process contributes to Bcl11b‐TAD formation and strengthens the interactions between enhancers and promoters.^245^

## 7 3D GENOME STRUCTURAL VARIATIONS AND DISEASE

The 3D genome structure is pivotal in gene expression regulation. Pathological conditions can lead to extensive reorganization of the higher‐order chromatin structure, which affects the expression of functional genes. The switching of aberrant A/B compartments at the subchromosomal level is closely linked to the onset and progression of the disease. Chromatin structural variations (SVs) at the megabase scale modify the TAD structure and interactions between regulatory elements, resulting in abnormal gene expression levels. Additionally, the formation of disease‐specific chromatin loops alters enhancer–promoter interactions, exacerbating gene expression dysfunction and contributing to disease development. This section systematically reviews the dynamic reorganization of higher‐order chromatin structures at different levels during the onset and progression of diseases. This may provide valuable insights into the complex relationship between chromatin conformation and diseases (Table 1).

**TABLE 1:** 3D genome structural variations and disease.

Chromatin Structure	Gene Locus	Diseases	Pathogenic mechanism	Reference
Compartment	Sugcct Fgfr2	Nonalcoholic fatty liver disease	Approximately 33% of the genome undergoes A/B compartment conversion. Sugct and FGFR2 are activated in association with Non‐alcoholic fatty liver disease.	^251^
Compartment	WNT5A CDK44	Prostate cancer	The metastatic potential and invasiveness of prostate cancer are closely linked to dynamic genome alterations. Forty‐eight genes have shifted from B to A compartments and activated.	^252^
Compartment	HNF4A NR5A2	HBV infection	HBV DNA integration into the human genome leads to the remodeling of the 3D genome structure. During this process, dynamic changes occur in the A/B compartments, including HNF4A and NR5A2.	^256^
Topologically associating domain	Sox9	Pierre‐Robin sequence	Deletions of the long‐range enhancer clusters of Ec1.45 and/or Ec1.25 in the Sox9‐TAD result in decreased expression levels of Sox9 and cause.	^267^
Topologically associating domain	Ihh	Abnormal Ossification of the Skull	Deletions of enhancers i2–i9 decreased the expression levels of Ihh which lead to abnormal ossification of the skull. Whereas duplication of i2–i9 increased Ihh expression.	^281^
Topologically associating domain	HoxD	2q31 syndrome	Sequence duplications or deletions within HoxD‐TAD resulted in a reduction to similar levels of enhancer–promoter interactions.	^288^
Topologically associating domain	Sox9	Sex reversal/disorder of sex development	Deletion of the Sox9 upstream enhancer eSR‐A(Enh13) results in sex reversal, whereas a sequence duplication involving this enhancer results in a disorder of sex development.	^268^, ^277^
Topologically associating domain	Pmp22	Hereditary neuropathy/Charcot Marie tooth.	Deletion of Pmp22‐SE, an upstream enhancer of PMP22, leads to hereditary neuropathy. Sequence duplications involving this enhancer are associated with Charcot‐Marie‐Tooth disease.	^271^
Topologically associating domain	Pitx1	Hindlimb deformity	The enhancer Pen tissue specifically regulates the Pitx1 expression in hindlimb development and aberrant Pitx1 expression by Pen deletion results in hindlimb deformity.	^319^
Topologically associating domain	Epha4 Pax3	Brachydac‐tyly	A heterozygous deletion of 1.75‐1.9 Mb on 2q35 spanning the TAD boundaries of EphA4 and Pax3 resulted in TAD fusion. In this fused TAD, an enhancer originally regulating EphA4 interacts with the Pax3 promoter.	^258^
Topologically associating domain	MYC‐BDME	T‐cell acute lymphoblastic leukemia	Abnormal MYC transcript levels in T‐cell acute lymphoblastic leukemia patients result from MYC‐TAD fusion with adjacent TADs, causing aberrant MYC promoter interactions with enhancers BDME.	^293^
Topologically associating domain	CFTR‐WNT2	Intestinal neoplasia	A 121.1 kb heterozygous deletion on 7q31.2 located in the border between CFTR and WNT2. Consequently, the enhancer (located in introns 1, 10, and 11), which initially regulates the tissue‐specific expression of CFTR, interacts with the WNT2 promoter.	^297^
Topologically associating domain	CDKN2A MIR31HG	Pancreatic ductal carcinoma	The proto‐oncogene MIR31HG‐TAD is adjacent to CDKN2A‐TAD, and sequence deletions involving TAD boundaries can result in the fusion of the two TADs. The proto‐oncogene MIR31HG promoter is aberrantly regulated by the enhancer, resulting in an increase in its expression level.	^299^
Topologically associating domain	Sox9 Kcnj2	Cooks Syndrome	Duplications spanning the Sox9‐TAD and Kcnj‐TAD give rise to “neo‐TAD”., and result in an enhancer otherwise regulating Sox9 expression incorrectly interacting with the Kcnj2 promoter in the “neo‐TAD”.	^304^
Topologically associating domain	YPEL2 GDPD1	Retinitis Pigmentosa	Sequence duplications spanning YPEL2, GDPD1 result in the creation of neo‐TAD, and enhancers originally located in YPEL2‐TAD ectopically regulate GDPD1 expression.	^307^
Topologically associating domain	GPR101RBMX	X‐linked acrogigantism	A 600 kb sequence duplication on chromosome Xq26.3 led to a neo‐TAD. Within the neo‐TAD, the GPR101 promoter interacted with the enhancer eRBMX from within the TAD on the centromeric side and up‐regulated the GPR101 transcript levels.	^300^
Topologically associating domain	SHH MNX1	Complete pulmonary hypoplasia	Sequence duplications spanning the SHH‐TAD and telomeric TAD generated 2 consecutive neo‐TAD. In the neo‐TAD, MACS1, an enhancer that originally regulates the tissue‐specific expression of SHH interacts with the MNX1 promoter.	^301^
Topologically associating domain	EGFR LINC01446	Glioblastoma	In glioblastoma multiforme, a sequence duplication between ZINCO1446 and EGFR on 7p11.2 creates a neo‐TAD, resulting in EGFR being aberrantly activated.	^311^
Topologically associating domain	TFAP2A	Branchiooculofacial syndrome	An 89 Mb inversion with a breakpoint within TAD disconnects the TFAP2A gene from enhancers such as Enh100 and Enh105, which resulted in haploinsufficient expression of the TFAP2A gene in human neural crest cells.	^315^
Topologically associating domain	Pitx1	Liebenberg syndrome	Pitx1 is regulated by enhancers RA4 and RA5, and enhancer Pen regulates tissue‐specific expression of Pitx1. A fragment inversion of 113 Kb containing Pen and RA4 places pen in the position of RA4, causing Pitx1 to be transcriptionally activated erroneously by Pen in forelimb development.	^320^
Topologically associating domain	Wnt6 Epha4	F syndrome	The heterozygous inversion of 1.1 Mb sequence in 2q36 region crosses the Wnt6‐TAD and Epha4‐TAD boundary caused the Epha4 enhancer to incorrectly regulate wnt6 expression.	^258^
Topologically associating domain	PRDM6SNCAIP	Medulloblastoma	The PRDM6 gene, situated at 5q23, is located approximately 600 kb downstream from SNCAIP. Owing to chromosomal inversion, the super‐enhancer initially responsible for regulating SNCAIP interacts with PRDM6.	^324^
Topologically associating domain	MEF2C	5q14.3 microdeletion syndrome	A complex translocation of 1Mb between 5q14.3 and 3q24 results in a disrupted TAD structure that disconnecting MEF2C from its cognate enhancers.	^329^
Topologically associating domain	Kcnj2 Kcnj16	Cooks syndrome	The translocation on chr17 disrupts the interaction of the KCNJ2 promoter with its cognate enhancer IMR90.	^312^
Topologically associating domain	GATA2 EVI1	Acute myeloid leukemia	Translocations between 3q21 and 3q26.2 have been observed in patients with acute myeloid leukemia, repositioning an enhancer located upstream of the GATA2 gene within the telomeric side TAD.	^335^
Topologically associating domain	MYB QKI	Angiocentric glioma	Translocations between 2q35 and 13q14 resulting in interaction of the FOXO1 enhancer B116Z with the Pax3 promoter have been observed in rhabdomyosarcoma cell lines.	^337^
Topologically associating domain	CCND1IGH	Malignant B cell lymphomas	CCND1 expression levels are increased more than 500‐fold in malignant B cell lymphomas. The t(11; 14) (q13; q32) causes a superenhancer that should intrinsically regulate IGH expression to interact with the CCND1, ultimately leading to a significant increase in CCND1 expression levels.	^333^
Chromatin loop	DDX60L CXXL13	Systemic lupus erythematosus	In Systemic lupus erythematosus patients, 391 disease‐specific chromatin loops are present, encompassing crucial inflammation‐ and immunity‐related genes, including DDX60L and CXCL13.	^373^
Chromatin loop	NPPA NPPB	Dilated cardiomyopa‐thy	During Dilated cardiomyopathy development, enhancer–promoter loop dynamic remodeling occurs extensively across the genome in response to rapid transcription.	^376^
Chromatin loop	LIPC	Pancreatic cancer	In metastatic pancreatic cancer cells, the number of chromatin loops increases LIPC expression is modulated by Enhancer 3 and Enhancer 4, while tissue‐specific chromatin loops form progressively during pancreatic cancer distant metastasis.	^378^
Chromatin loop	MYCN	Acute myeloid leukemia	Disease‐specific chromatin loop formation has been observed in AML, involving oncogenes. The specific interaction between the MYCN promoter and enhancers situated 650 kb downstream is related to AML onset.	^379^

### 7.1 Compartment and disease

The abnormal conversion of A/B compartments under pathological conditions may result in dysregulated gene expression, playing a crucial role in the onset and progression of diseases.^246^ Nonalcoholic fatty liver disease (NAFLD) is a chronic liver disease primarily observed in obese individuals, characterized by hepatic fat accumulation.^247^ NAFLD increases the risk of liver cirrhosis and hepatoma.^248^ Studies on NAFLD‐induced mice have revealed that around 33% of the genome undergoes A/B compartment conversion. Sugct and Fgfr2 are examples of genes transferred to the A compartment, linked to lipid metabolism disorders and tumor development potential, respectively.^249^, ^250^, ^251^ This finding enhances our understanding of NAFLD pathogenesis, disease progression, and the pathological mechanisms that make patients susceptible to liver cancer.^251^ The metastatic potential and invasiveness of prostate cancer are also intimately connected to dynamic genome alterations. Recent in vitro investigations on prostate cancer metastasis have revealed that during cancer progression, there is a widespread genome compartment conversion, which results in significant changes in the nuclear chromatin activation environment, leading to increased mixing and interaction in the A compartment.^252^ Forty‐eight genes have been transferred from transcriptionally repressed B compartments to A compartments and activated, including genes associated with prostate cancer progression and poor prognoses, such as WNT5A, CDK44, and TMPRS22. These research findings reveal potential key factors in the pathogenesis and progression of prostate cancer.^252^

Additionally, viruses can reshape the host's 3D genome structure, which affects gene expression and is closely linked to disease progression. The Hepatitis B virus (HBV) is a leading cause of hepatocellular carcinoma.^253^ Integration of HBV DNA into the human genome results in the remodeling of the 3D genome structure.^254^, ^255^ During this process, there are dynamic changes in the A/B compartments, mainly in the genomic regions on chromosomes 9, 13, and 21. Most genes are moved from transcriptionally repressed B compartments to A compartments, where they are subsequently activated.^256^ These regions have many transposable elements that promote viral DNA replication. A smaller subset of regions moves from the A compartment to the B compartment, including enhancers and genes associated with the regulation of inflammatory responses.^256^ Research on human cells infected with SARS‐CoV‐2 shows that about 30% of the genome undergoes compartment switching, which is accompanied by a decrease in chromatin interaction levels within the A compartment. This may be linked to the high incidence of acute sequelae resulting from SARS‐CoV‐2 infection.^257^

### 7.2 TAD and disease

**FIGURE 4:**
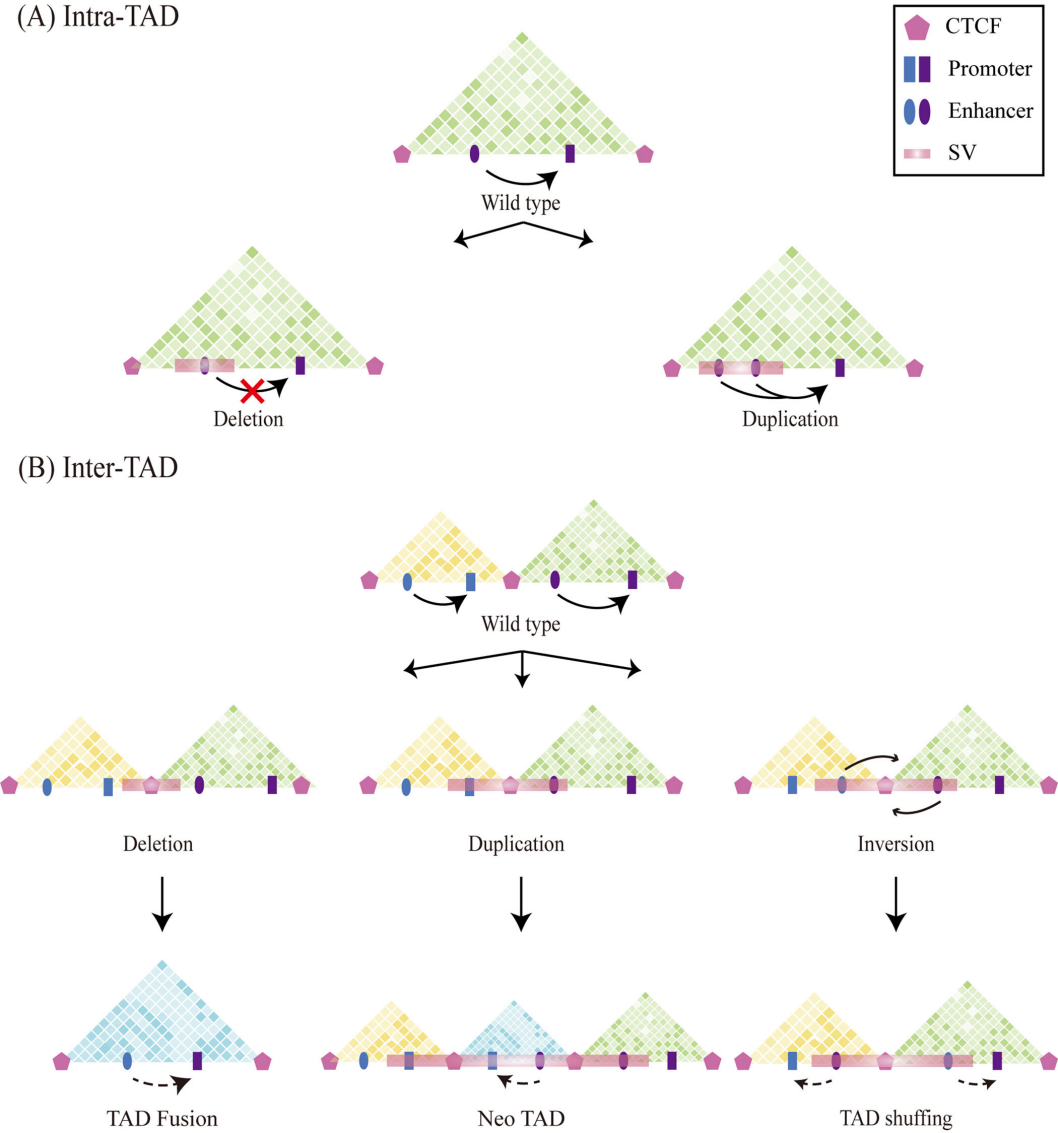
Structural variation located between TAD. (A) Sequence deletions or duplications within TADs primarily affect regulatory functions by altering the number of enhancers and causing abnormal expression of target genes. (B) Sequence deletions at the boundaries result in the merging of adjacent TADs into one, forming a new TAD, referred to as “TAD fusion.” Duplication of DNA sequences containing boundary elements can create a “neo‐TAD.” The “neo‐TAD” is situated between the original TADs, wherein the interacting enhancers and promoters originate from different initial TADs, and their interactions do not interfere with the enhancer–promoter interactions within the original TADs. Chromosomal inversions occurring between adjacent TADs alter the position and/or orientation of DNA segments, placing genes and/or regulatory elements in different chromosomal contexts, and causing pathological effects known as “TAD shuffling”.

Approximately 5% of the human genome exhibits structural variation.^258^ SVs include both balanced and unbalanced rearrangements, which lead to altered expression levels of functional genes by impacting the TAD structure. This results in aberrant enhancer–promoter interactions, contributing to the development of numerous human diseases (Table 1).^259^ Copy number variation comprises chromatin deletions and duplications, whereas inversions and translocations represent balanced rearrangements.^25^ This process is distinct from chromatin remodeling during cell cycle progression, which occurs in the nucleus without concomitant changes.^173^, ^260^ Copy number variants within the same TAD can alter gene expression by influencing the amount of regulatory elements or the frequency of interactions.^261^ Structural variations between TADs can affect gene expression by disrupting enhancer–promoter interactions or creating ectopic interactions (Figure 4).

Additionally, recent studies have shown that structural variation is a significant mechanism contributing to cancer.^262^, ^263^ Genomic rearrangements cause transcriptional dysregulation of proto‐oncogenes and oncogenes by changing interactions between cis‐regulatory elements in somatic cells, ultimately leading to abnormal proliferation and differentiation of somatic cells.^264^, ^265^, ^266^

#### 7.2.1 Deletions and duplications within TADs lead to diseases

Copy number variations within TADs significantly modulate gene expression by changing the quantity of regulatory elements or the frequency of interactions between them. Such changes can cause the dysregulation of gene expression, contributing to various human diseases and developmental disorders (Figure 4A).^267^, ^268^, ^269^, ^270^, ^271^, ^272^

Sox9 is located within a 2 Mb TAD,^273^, ^274^ containing multiple tissue‐specific enhancers that participate in mammalian sex determination, craniomaxillofacial development, and chondrogenesis.^268^, ^275^, ^276^, ^277^ Located 1.45 and 1.25 Mb upstream of the Sox9 gene, remote enhancer clusters Ec1.45 and Ec1.25 regulate its tissue‐specific expression in the mandibular process and the first branchial arch region.^267^ Deletion of Ec1.45 and/or Ec1.25 in the Sox9‐TAD leads to reduced Sox9 expression levels, causing Pierre–Robin sequence,^267^ a group of craniomaxillofacial developmental malformations such as mandibular dysplasia, cleft palate, and tongue recession.^278^, ^279^ Specifically, deletion of Ec1.45 in the human genome can cause a decrease of over 50% in the expression level of Sox9,^267^ whereas simultaneous deletion of both appears to have a more substantial impact on Sox9 expression.^280^

The Ihh gene participates in skeletal development, and its expression is regulated by at least nine enhancers (i1–i9) located within Ihh‐TAD.^281^ Deletion of Ihh in mouse models causes joint fusion and skeletal shortening,^282^ whereas Ihh duplication is associated with finger deformities and premature closure of cranial sutures.^283^, ^284^ Deleting enhancers i2–i9 within Ihh‐TAD in mice results in a 98% reduction of Ihh mRNA expression levels, leading to abnormal cranial ossification, reduced bone cortex, and shortened extremities.^281^ In contrast, duplication of i2–i9 enhances Ihh‐TAD endointeraction, increasing the expression of Ihh to five‐fold in the head and limbs, which results in premature closure of the cranial suture and syndactyly.^281^

It is worth noting that not all intra‐TAD fragment duplications result in increased enhancer–promoter interactions and target gene expression levels within the TAD. Alterations in enhancer–promoter distances can influence interaction frequency and, as a result, impact gene expression.^285^ 2q31 syndrome, characterized by facial malformations, mental retardation, and limb deformities,^286^ can result from sequence duplication or deletion within HoxD‐TAD.^287^, ^288^ This occurs because sequence duplication increases the distance between the enhancer and gene, which weakens the interaction between the HoxD promoter and its enhancer, resulting in a 50% downregulation of the HoxD gene cluster transcript levels. In this case, sequence duplication within HoxD‐TAD leads to similar changes in gene expression levels as deletion.^288^

#### 7.2.2 Deletions between TADs lead to diseases

Copy number variations across TAD boundaries can disrupt chromosomal rearrangements of boundary elements. Deletions at the boundaries can merge adjacent TADs, resulting in a “TAD fusion”,^258^ and can lead to abnormal gene expression levels as enhancers from different TADs interact with promoters (Figure 4B).^289^ Structural variation has been identified as a crucial mechanism underlying cancer development.^262^, ^263^ Genomic rearrangements can cause transcriptional dysregulation of proto‐oncogenes and oncogenes by altering interactions between cis‐regulatory elements in somatic cells, leading to abnormal proliferation and differentiation.^264^, ^265^, ^266^

##### Congenital diseases

An example of chromosomal rearrangement that impacts gene expression is the Wnt6/Ihh/Epha4/Pax3 locus located on chromosome 2q35‐36. A heterozygous deletion of 1.75–1.9 Mb in the 2q35 region results in short‐fingered malformation in humans and mice. This deletion disrupts the TAD boundary between Epha4 and Pax3, leading to TAD fusion and producing an 800 kb fused TAD.^258^ Within this fused TAD, the enhancer that initially regulated Epha4 interacts with the Pax3 promoter, causing an increased expression level of Pax3 and a decreased expression level of Epha4, ultimately leading to the development of short‐fingered malformations.

Not all deletions across TAD boundaries lead to TAD fusion. In the mouse genome, adjacent motifs Sox9‐Kcnj show that deleting only the CTCF locus at the boundary does not result in TAD fusion. TAD fusion occurs only after deleting all four CTCF loci within the TADs. Only deleting all four CTCF loci within the TADs leads to TAD fusion, but it does not significantly affect gene expression.^290^ The limited impact on gene expression resulting from small deletions may be due to the redundancy of CTCF sites in the TADs. This redundancy mechanism helps maintain the structural and functional stability of TADs and ensures precise gene expression.

##### Cancers

The oncogene MYC is a critical downstream target gene of Notch1 signaling.^238^, ^291^ Its expression levels are frequently elevated in patients with T‐cell acute lymphoblastic leukemia (T‐ALL).^292^ Recent studies have shown that deletions of TAD boundaries in T‐ALL patients result in the fusion of MYC‐TAD with adjacent TADs, leading to abnormal interactions between the enhancer BDME/BENC and the MYC promoter within the fused TAD. This results in increased expression of MYC and the development of T‐ALL.^293^

CFTR and WNT2 genes are located in adjacent TADs on chromosome 7. Research on patients with intestinal neoplasia pedigrees has shown that a 121.1 kb heterozygous deletion on 7q31.2 disrupts the border between CFTR and WNT2 and deletes the CFTR promoter sequence.^294^ Consequently, the enhancer located in introns 1, 10, and 11 that originally regulates the tissue‐specific expression of CFTR interacts with the WNT2 promoter, resulting in increased levels of WNT2 expression and decreased levels of CFTR expression in this pedigree.^294^, ^295^, ^296^ This dysregulation is associated with the development of intestinal adenocarcinoma and small intestinal neuroendocrine tumors.^295^, ^297^

The majority of patients with pancreatic ductal carcinoma present a homozygous deletion of CDKN2A.^298^ Recent studies have shown that MIR31HG‐TAD is adjacent to CDKN2A‐TAD in various pancreatic ductal carcinoma cell lines. Deletions in the boundary regions of the two TADs result in their fusion. In the fused TADs, the MIR31HG promoter is abnormally regulated by the enhancer, leading to increased expression levels.^299^

#### 7.2.3 Duplications between TADs lead to diseases

Duplication of DNA sequences containing boundary elements can result in the formation of “neo‐TADs”,^300^, ^301^, ^302^ which are located between the original TADs. The enhancers and promoters within these “neo‐TADs” originate from different initial TADs, but their interactions do not interfere with the enhancer–promoter interactions in the original TADs (Figure 4B).

##### Congenital diseases

Cooks syndrome is linked to chromosomal duplications and is characterized by nail hypoplasia and short‐fingered malformations.^303^ The duplication of the Sox9‐TAD and Kcnj‐TAD creates a “neo‐TAD”, as evidenced by RNA‐seq results from mouse limbs at various developmental stages that show no changes in the expression levels of Sox9 and Kcnj16, but an increase in Kcnj2 expression.^304^ This rise in Kcnj2 expression is a result of misinteraction between the enhancer that originally regulated Sox9 expression and the Kcnj2 promoter within the “neo‐TAD”.

Retinitis pigmentosa is a common inherited retinal disease characterized by progressive peripheral vision loss and night blindness, which can lead to blindness in severe cases.^305^ The disease is associated with a chromosome 17q22 duplication spanning the YPEL2 and GDPD1 genes. YPEL2 is highly expressed in the brain and retina,^306^ whereas GDPD1 encodes glycerophosphodiesterase and is predominantly expressed in the brain and testis.^307^ This duplication leads to the formation of new interaction domains, where enhancers originally located in YPEL2‐TAD regulate GDPD1 expression ectopically.^307^ Elevated GDPD1 expression levels can cause dysregulation of lipid metabolism, a pathogenic factor contributing to photoreceptor cell inactivation and the development of retinitis pigmentosa.^308^, ^309^, ^310^

##### Cancer

EGFR and LINC01446 are situated in adjacent TADs on chromosome 7p11.2. In cell lines derived from glioblastoma patients, a sequence duplication was observed between EGFR and LINC01446, generating a “neo‐TAD” between the two adjacent TADs. Within the “neo‐TAD,” the enhancer initially intended to regulate LINC01446 expression aberrantly interacts with the promoter responsible for regulating EGFR expression, leading to an increase in EGFR expression levels. This is one of the pathological mechanisms that contribute to the development of glioblastoma.^311^

#### 7.2.4 Inversion between TADs leads to diseases

Balanced chromosomal rearrangements, including inversions and translocations, occur between spatially adjacent or separated TADs, changing the position and/or orientation of DNA segments.^27^ Balanced rearrangements cause pathological effects by placing genes and/or regulatory elements in different chromosomal environments, a phenomenon known as “TAD shuffling”.^312^ TADs that experience balancing rearrangements result in abnormal gene expression due to disrupted enhancer–promoter interactions, enhancer hijacking effects, or positional effects (Figure 4B).^27^

##### Congenital diseases

The mechanisms described above can explain the etiology of chromosomal inversions causing congenital diseases such as branchiooculofacial syndrome and Liebenberg syndrome. Branchiooculofacial syndrome is a rare developmental defect caused by heterozygous deletions or mutations in the TFAP2A gene.^313^, ^314^ In patients with branchiooculofacial syndrome, an 89Mb inversion was found with a breakpoint located in the TAD, which disconnects the TFAP2A gene from enhancers such as Enh100 and Enh105, leading to haploinsufficient expression of TFAP2A in human neural crest cells.^315^

Liebenberg syndrome is characterized by a heterozygous leg‐arm transformation, where the upper limb exhibits morphological features typically seen in the lower limb.^316^, ^317^ Pitx1 is expressed in the hindlimbs, pituitary gland, and first‐gill arch, and gene rearrangements involving Pitx1 are associated with the disorder.^318^, ^319^ During limb development, Pitx1 is regulated by enhancers RA4 and RA5, in both the anterior and posterior limbs.^320^ However, enhancer Pen specifically regulates Pitx1 expression in the hindlimbs, and there is no interaction with Pitx1 during forelimb development.^318^, ^320^ In Liebenberg syndrome, an inversion of a 113‐kb fragment containing enhancers Pen and RA4 results in the placement of Pen in the position of RA4. This misplacement causes Pitx1 to be activated by Pen during forelimb development, leading to the development of phenotypes such as radial curvature, patellar heterotaxy, and shortened ulnar hawk in adult mice.^320^

##### Cancers

Medulloblastoma is a highly malignant tumor of the central nervous system that commonly occurs in children.^321^, ^322^, ^323^ Recent studies suggest that chromosomal inversions may significantly contribute to the development of this disease.^324^ The PRDM6 gene, encoding a histone transferase, is located approximately 600 kb downstream from SNCAIP at 5q23. Chromosomal inversion leads to the interaction between the super‐enhancer responsible for regulating SNCAIP and PRDM6, resulting in a significant increase of approximately 20‐fold in PRDM6 expression levels.^262^, ^324^

#### 7.2.5 Translocations between TADs lead to diseases

##### Congenital diseases

Chromosomal translocations can also cause developmental defects through pathological mechanisms akin to inversions. MEF2C is considered one of the crucial pathogenic genes in the 5q14.3 microdeletion syndrome,^325^, ^326^ which is associated with developmental brain malformations, epilepsy, and intellectual deficits.^327^, ^328^ A 1 Mb complex translocation between 5q14.3 and 3q24 disrupts the TAD structure, disconnecting MEF2C from the enhancer that regulates its expression.^329^ Chromosomal translocations reduce MEF2C expression to 50% compared with controls,^329^ a level comparable to that observed in humans with MEF2C heterozygous deletions.^330^

##### Cancers

A chromosomal translocation involving 11q13 and 14q32 has been identified in multiple myeloma and mantle cell lymphoma, and is associated with the CCND1 and IGH gene loci.^331^ The proto‐oncogene CCND1, situated on chromosome 11, encodes a cell cycle protein, while the IGH gene is located on chromosome 14.^332^ This translocation allows the superenhancer, which initially regulates IGH, to aberrantly activate CCND1 expression, leading to an up to 500‐fold increase in its expression levels.^333^

Acute myeloid leukemia (AML) is linked to aberrant expression of the stem cell factor EVI1. Chromosomal translocations involving 3q21 and 3q26.2 have been observed in AML patients.^334^ These rearrangements permit the upstream enhancer of the GATA2 gene to relocate and aberrantly interact with the EVI1 promoter, leading to increased EVI1 expression and GATA2 haploinsufficiency. As a result, hematopoietic stem cell growth and differentiation are impeded, contributing to the development of AML.^335^

Angiocentric glioma, a low‐grade malignant glioma, is associated with the MYB‐QKI rearrangement.^336^ The proto‐oncogene MYB and the oncogene QKI reside at 6q23.3 and 6q25.3, respectively. Chromosomal translocations lead to the enhancers Q3SE1 and Q3SE2, which initially regulate QKI transcription, aberrantly activating the proto‐oncogene MYB expression. Consequently, the fusion protein MYB‐QKI, a proto‐oncoprotein, contributes to the development of angiocentric glioma.^337^

Chromatin remodeling can contribute to cancer development by altering the local 3D genomic structure. Human papillomavirus (HPV) genes can integrate into the human genome, facilitating cervical cancer development through TAD structure remodeling.^338^, ^339^ PEG3 and CCDC16, genes co‐located within the same TAD on chromosome 19, are impacted by this alteration.^340^, ^341^ HPV reshapes the TAD structure, dividing it into two unequal TADs. As a result, enhancers initially regulating PEG3 aberrantly activate the proto‐oncogene CCDC16 expression, contributing to cervical cancer development.^338^

#### 7.2.6 Abnormalities in structural proteins lead to diseases

The “loop extrusion” model, mediated by CTCF, cohesin, and their regulators, effectively elucidates TAD formation and remote enhancer–promoter interactions within TAD.^342^ Previous research has demonstrated distinct functions of cohesin and CTCF in TAD formation.^78^, ^79^ Abnormalities in cohesin or CTCF function can result in aberrant gene expression due to alterations in higher chromatin structure. Mutations in genes encoding cohesin subunits or regulatory factors can disrupt TAD formation and chromatin interactions, affecting normal gene expression.^343^ These mutations, which impact cohesin activity and function, are termed “cohesinopathies”.^344^ Cornelia de Lange syndrome (CdLS) is characterized by intellectual disability, microcephaly, growth retardation, and upper limb deformities.^345^ CdLS pathogenesis is linked to cohesin dysfunction.^344^, ^345^ NIPBL mutations are found in 65% of CdLS patients.^346^, ^347^ Normally, the cohesin loader NIPBL introduces cohesin into the promoter of highly expressed genes,^84^, ^348^, ^349^ facilitating its movement along chromatin fibers.^87^, ^350^ In primary fibroblasts derived from CdLS patients, cohesin is still loaded at specific sites, but its chromatin fiber binding stability is reduced.^351^ The NIPBL mutation decreases cohesin mobility and ultimately affects the DNA loop extrusion process and TAD formation.^351^

CTCF is involved in forming chromosomal higher‐order structures and plays a crucial role in cell differentiation and apoptosis.^352^, ^353^, ^354^ Located at TAD boundaries, CTCF avoids ectopic contact with enhancers while promoting high‐frequency enhancer–promoter interactions within the TAD. However, CTCF is highly sensitive to methylation levels,^355^, ^356^ and abnormally elevated methylation levels at CTCF‐DNA binding sites cause disruption of the TAD boundaries,^357^ leading to a decrease in enhancer–promoter interactions within the TAD and an increase in inter‐TAD interactions.^78^ This mechanism was first identified in the study of isocitrate dehydrogenase (IDH) mutant gliomas. IDH mutant glioma patients have increased levels of DNA methylation,^357^, ^358^ resulting in ectopic interaction between the enhancer located 50 kb upstream of FIP1L1 and the proto‐oncogene PDGFRA promoter, leading to a three‐fold increase in the expression level of proto‐oncogene PDGFRA and promoting glioma cell proliferation.^359^ Similarly, in regions of pathogenic short tandem repeats, local DNA methylation levels elevate and affect local CTCF binding sites.^360^, ^361^ Short tandem repeats comprise repeats of three or more base pairs in a DNA sequence. They make up approximately 1% of the human genome and are generally non–pathogenic.^362^ Recent Hi‐C data suggested that in various congenital disorders, such as fragile X syndrome, Friedreich's ataxia, and Huntington's disease, pathogenic short tandem repeat sequences are located at the TAD boundary.^363^, ^364^, ^365^, ^366^ These pathogenic short tandem repeat regions have abnormally elevated local methylation levels and alter the TAD boundaries, contributing to many congenital disorders.^367^, ^368^ For example, FMR1 has been identified as the pathogenic gene in fragile X syndrome.^369^ Disruption of the FMR1‐TAD boundary due to increased local methylation in the genome of Fragile X syndrome patients results in disrupted FMR1 interaction with telomere orientation cognate enhancers and decreased FMR1 expression.^370^

### 7.3 Chromatin loop and disease

During disease development, chromatin loops experience reprogramming in a cell‐specific manner, regulating gene expression. Systemic lupus erythematosus (SLE) is an autoimmune disease frequently involving multiple organs.^371^ In CD4^+^ T cells derived from SLE patients, 391 disease‐specific chromatin loops are present, encompassing crucial inflammation‐related and immunity‐related genes, including DDX60L and CXCL13.^372^, ^373^ Notably, the DDX60L locus contains two disease‐specific chromatin loops, whose formation is associated with histone modifications in the promoter region mediated by transcription factor SPI1.^372^, ^373^

Dilated cardiomyopathy (DCM) is a leading cause of heart failure.^374^, ^375^ Recent studies have revealed that during DCM development, enhancer–promoter loop dynamic remodeling occurs extensively across the genome in response to rapid transcription under cardiac stress conditions.^376^ Chromatin loops reprogramming, directly driven by transcription factor HAND1, results in elevated expression levels of DCM‐associated pathogenic genes.^376^ For instance, the NPPA‐AS1 promoter possesses enhancer functions, interacting with NPPA and NPPB promoters during DCM development and leading to the co‐transcription of NPPA and NPPB.^376^

Distant metastasis in pancreatic cancer significantly contributes to its poor prognosis.^377^ Recent investigations suggest that pancreatic cancer distant metastasis correlates with epigenetic alterations.^378^ In metastatic pancreatic cancer cells, the number of chromatin loops increases, along with the emergence of cell‐specific chromatin loops. LIPC, a gene promoting pancreatic cancer metastasis, is implicated in tumor cell migration and invasion.^378^ LIPC expression is modulated by Enhancer 3 and Enhancer 4, while tissue‐specific chromatin loops form progressively during pancreatic cancer distant metastasis, enhancing LIPC expression.^378^ Additionally, disease‐specific chromatin loop formation has been observed in AML, involving oncogenes such as MYCN, WT1, and RUNX1.^379^ For example, a specific interaction between the MYCN promoter and enhancers situated 650 kb downstream is related to AML onset.^379^

## 8 CONCLUSION AND PERSPECTIVE

The 3D genome structure and its functions have long been a focal point. In recent years, significant advances have been made in this area due to the rapid development of chromatin conformation capture techniques and super‐resolution fluorescence imaging technologies. In this review, the structural hierarchy of the 3D genome, the effect and mechanisms of cis‐regulatory element interactions in the 3D genome for regulating spatiotemporally specific gene expression, the role and mechanisms of dynamic changes in 3D chromatin conformation during embryonic development, and the pathological mechanisms of diseases such as congenital developmental abnormalities and cancer, which are attributed to alterations in 3D genome organization and aberrations in key structural proteins, were systematically discussed. In summary, the 3D genome structure plays crucial roles in cell differentiation and disease development by regulating spatiotemporal gene expression, which may offer some clues for precise diagnosis and treatment of related diseases.

Nevertheless, further research is needed to understand the fundamental principles of 3D genome organization and the relationship between 3D genome structure and spatiotemporal gene expression. The recent development of single‐cell chromatin conformation capture techniques and genome architecture mapping technologies has enabled a more in‐depth exploration of the structural and functional features of the 3D genome. This also includes the mechanisms by which chromatin higher‐order structures regulate cell‐type‐specific gene expression, thereby shedding light on the impact of the genome's spatial organization on cell differentiation and fate determination.

Furthermore, SVs and abnormalities in structural proteins can influence the function of cis‐regulatory elements, leading to atypical gene expression and, consequently, various diseases. Advancements in chromatin conformation capture techniques and transcriptomics will facilitate a deeper understanding of the pathological mechanisms underlying developmental defects and cancer. This will provide new theoretical insights and research directions for prenatal screening and precision diagnosis and introduce novel therapeutic targets for treating a more comprehensive range of congenital diseases and malignant tumors.

## AUTHOR CONTRIBUTIONS

H.L. wrote the manuscript and reviewed the literature. H.T. wrote the manuscript and drew the pictures. M.Y. reviewed the literature and drew the pictures. G.L. wrote the manuscript and reviewed the literature. Q.B. selected and revised the article. G.D. selected and revised the article. D.W. selected and proofread the article. J.D. selected, proofread, and revised the article. All authors read and approved the final manuscript.

## CONFLICT OF INTEREST STATEMENT

The authors declare that they have no conflict of interest.

## ETHICS STATEMENT

Not applicable.

## Data Availability

Data are available upon request from the authors.
